# Fellow-Workers and the Roll of Honour

**Published:** 1915-04-17

**Authors:** 


### ROUND THE WORLD.

[The Editor invites Early Information and Contributions Marked "Fellow-Workers."]

### Fellow-Workers and the Roll of Honour.

Mr. Herbert John Paterson, M.C. Cantab., F.R.C.S. Eng., who is in charge of the Queen Alexandra Hospital for Officers at Highgate, is one of our foremost authorities on gastro-intestinal surgery. At the London Temperance Hospital, where he has been senior surgeon since the retirement of Sir William Collins, Mr. Paterson has carried out important investigations in regard to the surgery of the stomach and appendix. A brilliant student of "Bart.'s," it is to be noted that he has an enthusiastic following amongst American surgeons, many of whom have visited his clinic.

The late Lieutenant Pierce Michael Joseph Power, R.A.M.C., killed in France, was an Irish student, who qualified by taking the conjoint diploma of the Irish Royal Colleges of Physicians and Surgeons in 1910. He also took the Dublin L.M., and two years after qualifying entered the medical department of the Army.

The series of post-graduate lectures for nurses arranged by the joint War Committee of the British Red Cross Society and the Order of St. John of Jerusalem, but since cancelled, were to have been given by Dr. D. Forsyth and Mr. P. Turner.

Dr. David Forsyth is a member of the staff of Charing Cross Hospital, where he holds the posts of physician to out-patients and lecturer on therapeutics and pharmacology. A Guy's Hospital man, he was house physician and demonstrator of physiology there in his early years. After qualifying at the Conjoint Board's examinations Dr. Forsyth became an M.D. Lond., and also took the degree of D.Sc. at the home university; having become a member of the Royal College of Physicians, he was elected to the fellowship in 1910. His interest in children's diseases is well known, and his post of physician to the Evelina Hospital has given him opportunities for making important observations in this respect.

Mr. Philip Turner, M.S. Lond., F.R.C.S. Eng., is one of the assistant surgeons at Guy's, where he has had a brilliant career. Entering the medical school with a scholarship, he qualified in 1897, and subsequently took honours at the M.B. Lond. examination; also sitting for the B.Sc. degree, where he again scored honours. Having been resident medical officer to the private wards at Guy's Mr. Turner became surgeon in charge of the ear department there and assistant surgeon to the Belgrave Hospital for Children, responsible appointments which he has since given up. He is the author of a small work on the skeleton entitled "The Pocket Osteology."

Great credit is due to the distinguished ladies in medical charge of the French Red Cross Hospital at Royaumont for the way in which they have discharged their onerous duties; their work having attracted the favourable attention of the French Government. At the institution named are working Miss M. H. F. Ivens, Dr. Agnes Savill, Miss D. Hancock, and Miss W. Ross.

Miss Mary Hannah Frances Ivens, M.B., M.S. Lond., is one of the most brilliant representatives of the London School of Medicine for Women in practice. A prominent practitioner in Liverpool, where she holds the posts of honorary medical officer for diseases of women at the Stanley Hospital and honorary surgeon to the Samaritan Hospital, Miss Ivens is now directing surgeon at the Royaumont Red Cross Hospital. In the course of an exceptionally successful student career she won the university gold medal and scholarship in obstetric medicine, with honours in medicine and forensic medicine at the M.B. Lond., as well as honours at the B.S. Lond. Her past appointments include those of house surgeon at the Royal Free Hospital and out-patient surgeon to Clapham Maternity Hospital.

Dr. Agnes Savill is a London physician who has made a name for herself in more than one direction, her chief studies having been dermatology and electrotherapy. An M.A. of St. Andrews University, she qualified at Glasgow in 1898, and subsequently became an M.D. of the University there, and a member of the Irish Royal College of Physicians. At one time she was physician to St. John's Hospital for Skin Diseases, and to the skin department of the Women's Hospital for Children, whilst her present appointments include that of assistant physician to the London Skin Hospital in Fitzroy Square. Dr. Agnes Savill has written extensively on electrical treatment, and particularly of the uses of $x$-rays in connection with skin diseases.

Dr. Gordon M. Holmes, who is to be congratulated on his promotion to the honorary rank of major in the R.A.M.C., is one of our foremost neurological investigators. A Dublin student and an M.D. of the University, he was last year made an F.R.C.P. Lond. His present appointments include those of assistant physician to Charing Cross Hospital, and to the National Hospital for Paralysis and Epilepsy in Queen Square.

Two members of the medical service in West Africa were lost in the disaster to the *Falaba*, which went down after a submarine attack in the Irish Sea recently. These ill-fated officers were Mr. F. J. A. Baldwin and Mr. A. W. H. Grant.

Mr. Francis John Augustus Baldwin qualified at the Apothecaries' Hall in 1891, and a few years later secured the diploma of the Conjoint Board. A London Hospital man, he decided to take up service in the Colonies, for which he better fitted himself by taking the certificate of the London School of Tropical Medicine. Having joined the West African Staff, he had lately been working in South Nigeria.

His equally unfortunate colleague, Mr. Alexander William Harvey Grant, was considerably junior in West Africa. A student of Charing Cross Hospital, he became an L.S.A. in 1901, and went to South Africa as civil surgeon to the Field Force during the latter stages of the Boer War. Subsequently Mr. Grant was for a time in the asylum service, holding the post of assistant medical officer both at the Brecon and Radnor Asylum, and at the Three Counties institution at Hitchin.

Sadly enough, the medical officer of the *Falaba* seems also to have gone down with his ship. Mr. Simon Vincent Daly was a student of the Irish schools, who qualified some thirty years ago; he was well known in Monkstown, Cork County.

